# Microsatellite instability combined with PD-L1 expression in prognosis of gastric cancer: an integrated analysis based on individual patient data

**DOI:** 10.3389/fonc.2026.1900024

**Published:** 2026-09-17

**Authors:** Yajing Feng, Mengmeng Yin, Junhui Chai, Jicun Zhu, Yuehua Zhang, Kaijuan Wang, Fujiao Duan

**Affiliations:** 1 Department of Nosocomial Infection Management, The First Affiliated Hospital of Zhengzhou University, Zhengzhou, Henan, China; 2 Department of Medical Research Office, The Affiliated Cancer Hospital of Zhengzhou University & Henan Cancer Hospital, Zhengzhou, Henan, China; 3 College of Public Health, Zhengzhou University, Zhengzhou, Henan, China; 4 Henan Key Laboratory of Tumor Epidemiology, Henan International Joint Laboratory of Tumor Biomarkers and Molecular Imaging, Zhengzhou University, Zhengzhou, Henan, China

**Keywords:** gastric cancer, immunotherapy, meta-analysis, microsatellite instability, PD-L1, prognosis

## Abstract

**Objective:**

This study aimed to systematically assess the predictive value of microsatellite instability (MSI) combined with programmed death-ligand 1 (PD-L1) expression for prognosis and immunotherapy response in patients with gastric cancer (GC).

**Methods:**

An individual patient data (IPD) meta-analysis was performed in accordance with the PICOS framework and PRISMA 2020 guidelines. A comprehensive literature search was performed across English and Chinese databases to identify cohort studies or randomized controlled trials (RCTs) that simultaneously evaluated MSI status and PD-L1 expression, and reported overall survival (OS), disease-free survival (DFS) with 95% confidence intervals (CIs) were used as pooled effect measures. Fixed-effect or random-effects models were applied based on heterogeneity assessed by the *I*² statistic. Subgroup analyses were stratified by ethnicity, pathological stage, Lauren classification, chemotherapy timing, MSI detection platform, and PD-L1 antibody clone. The interactive effect of MSI-H and PD-L1 expression was evaluated using radar plots.

**Results:**

Nine studies involving 6,667 GC patients were included. For OS, MSI-H alone (HR = 0.500, 95%CI: 0.357-0.700, *P* < 0.001), and MSI-H/MSI-L combined (HR = 0.641, 95%CI: 0.437-0.939, *P* = 0.025) were associated with better outcomes than MSS; PD-L1 positivity predicted poorer OS (HR = 1.517, 95%CI: 1.077-2.137, *P* = 0.017); MSI-H+PD-L1 negative patients had the most favorable OS (HR = 0.380, 95%CI: 0.255-0.566, *P* < 0.001), while MSI-H+PD-L1 positive patients had poorer OS than MSS+PD-L1 negative patients (HR = 2.076, 95%CI: 1.238-3.479, *P* = 0.006). For DFS, MSI-H alone (HR = 0.506, 95%CI: 0.360-0.710, *P* < 0.001) and MSI-H/MSI-L combined (HR = 0.530, 95%CI: 0.402-0.698, *P* < 0.001) outperformed MSS, but PD-L1 expression showed no significant DFS association (HR = 1.189, 95%CI: 0.472-2.998, *P* = 0.709). Subgroup analyses identified TNM stage, Lauren classification, chemotherapy timing, and MSI detection method as significant effect modifiers (all interaction *P* < 0.05). Pooled immunotherapy data showed that MSI-H patients had higher ORR than MSS (OR = 3.580, *P* < 0.001), and MSI-H+PD-L1 positive patients achieved the highest response rates (OR = 4.100 vs. MSI-H+PD-L1 negative, *P* < 0.001). Sensitivity analysis confirmed result stability, and publication bias was minimal.

**Conclusion:**

MSI-H status is a robust favorable prognostic factor for GC, while PD-L1 positivity indicates adverse OS prognosis. The combination of MSI and PD-L1 expression enables refined prognostic stratification of GC patients.

## 1 Introduction

Gastric cancer (GC) remains one of the most prevalent malignant tumors globally, ranking fifth in incidence and third in mortality among all cancers (1). Despite advances in surgical resection, chemotherapy, and targeted therapy, the prognosis of GC patients remains unsatisfactory, with significant individual differences in treatment response and survival outcomes (2). The heterogeneous nature of GC at the molecular level highlights the urgency of identifying reliable biomarkers for prognostic stratification and treatment guidance (3).

Microsatellite instability (MSI), caused by defects in the DNA mismatch repair (MMR) system, is a key molecular characteristic of GC, accounting for 3.4%-38% of GC cases (4, 5). Previous studies have shown that MSI-high (MSI-H) GC patients have a relatively favorable prognosis and may benefit from immunotherapy (6, 7). Programmed death-ligand 1 (PD-L1), as an important immune checkpoint molecule, mediates tumor immune escape by binding to programmed death-1 (PD-1) on T cells (8). PD-L1 expression in GC tissues has been reported to be associated with poor prognosis, but its predictive value for immunotherapy response remains controversial (9, 10).

Recent studies have suggested that the combination of MSI status and PD-L1 expression may provide more comprehensive prognostic and predictive information than either marker alone (11, 12). However, existing studies have inconsistent results due to differences in sample size, detection methods, and interpretation criteria. Therefore, this integrated analysis based on individual patient data was conducted to systematically explore the combined role of MSI and PD-L1 in GC prognosis and immunotherapy response, aiming to provide a more reliable evidence-based basis for clinical practice.

## 2 Materials and methods

This study was fully designed and reported in accordance with the unified PICOS framework and PRISMA 2020 systematic review bias control guidelines, to reduce retrieval, screening and analytical bias throughout the whole research process.

### 2.1 Search strategy

A comprehensive literature search was performed in PubMed, Embase, Cochrane Library, Web of Science, Wanfang (Chinese), and CNKI (Chinese) from database establishment from database establishment to December 2025. The search terms included “gastric cancer”, “gastric carcinoma”, “microsatellite instability”, “MSI”, “programmed death-ligand 1”, “PD-L1”, “prognosis”, “survival”, “immunotherapy”, and “clinical response”. The search strategy was adjusted according to the characteristics of different databases. Duplicate records were removed through a two-stage strategy. Initial deduplication was performed automatically with EndNote X9 software, after which two researchers independently manually cross-checked the remaining records. To capture any eligible studies that might have been overlooked, we also conducted snowball reference tracking for all full-text articles and pertinent meta-analyses.

### 2.2 Inclusion and exclusion criteria

Inclusion criteria: (1) Cohort study or RCT; (2) Patients with pathologically confirmed GC; (3) Simultaneous detection of MSI status (MSI-H/MSI-L/MSS) and PD-L1 expression level, with clear detection methods and interpretation standards; (4) Reporting overall survival (OS), disease-free survival (DFS); (5) Providing available survival data (HR and 95%CI) or treatment response data (OR and 95%CI); (6) Chinese or English.

Exclusion criteria: (1) Study type: Review, case report, basic experimental study, or conference abstract without complete data; (2) Data defects: Unclear MSI/PD-L1 detection methods, missing outcome indicators, unavailable data, or duplicate publication; (3) Patient population: Patients with other malignant tumors, severe underlying diseases, or without receiving standard treatment (radical surgery/chemotherapy/immunotherapy); (4) Ununified detection standards: Unclear MSI instability site criteria or lack of PD-L1 combined positive score (CPS) interpretation standards.

### 2.3 Data extraction

Two researchers independently screened the literature according to the inclusion and exclusion criteria. Disagreements were resolved through discussion with a third researcher.

The extracted data included: (1) Basic study information: First author, publication year, region, study type; (2) Baseline characteristics of patients: Sample size, age, gender, TNM stage, Lauren classification; (3) MSI-related information: Detection method (PCR/IHC), diagnostic criteria, MSI-H incidence; (4) PD-L1-related information: Detection antibody, CPS cutoff value, positive rate; (5) Outcome indicators: HR and 95%CI for OS/DFS, OR and 95%CI for immunotherapy ORR; (6) Treatment-related information: Treatment regimen (surgery/chemotherapy/immunotherapy).

### 2.4 Quality evaluation

The Newcastle-Ottawa Scale (NOS) was used to evaluate the quality of cohort studies, with a score range of 0-9. Studies with a score ≥7 were considered high-quality studies. The Cochrane Risk of Bias Assessment Tool was used to evaluate RCTs, including random sequence generation, allocation concealment, blinding of participants and personnel, blinding of outcome assessment, incomplete outcome data, selective reporting, and other biases.

### 2.5 Statistical analysis

Stata 17.0 software was used for statistical analysis. Heterogeneity among studies was tested by the *I*² statistic. If *I*²<50%, it indicated low heterogeneity, and a fixed-effects model was used; if *I*²≥50%, it indicated moderate to high heterogeneity, and a random-effects model was used. OS and DFS were conducted according to MSI status, PD-L1 expression, and their combinations. Subgroup analyses were carried out by ethnicity, TNM stage, Lauren histotype, chemotherapy timing, MSI testing platform and PD-L1 antibody type. Sensitivity analysis was performed by sequentially excluding each study to test the stability of the results. Publication bias was evaluated by funnel plot and Egger’s test.

Statistical significance was defined as a two-sided *P*-value <0.05.

## 3 Results

### 3.1 Literature search results

A total of 797 studies were initially retrieved through database searching. After duplicate removal, title and abstract screening, and full-text eligibility assessment, 9 studies (7, 9, 12–18) involving 6,667 GC patients were finally included (**Figure 1**). Among the included studies, 7 were retrospective cohort studies, 1 was a prospective cohort study, and 1 was a *post-hoc* analysis of a RCT. The studies were conducted in multiple regions including China, South Korea, Italy, Finland, and international multi-center settings. The sample size of each study ranged from 100 to 2,341 cases. For MSI detection, 5 studies used PCR and 4 used IHC targeting mismatch repair (MMR) proteins, with MSI-H diagnostic criteria uniformly defined as ≥2 unstable loci (for PCR) or loss of ≥1 MMR protein (for IHC). PD-L1 detection antibodies included 22C3 (4 studies), SP142 (3 studies), and E1L3N (2 studies), with the combined positive score (CPS) cutoff value for PD-L1 positivity mainly set at ≥1. The detailed baseline characteristics of the included studies are summarized in **Table 1**.

**Figure 1:**
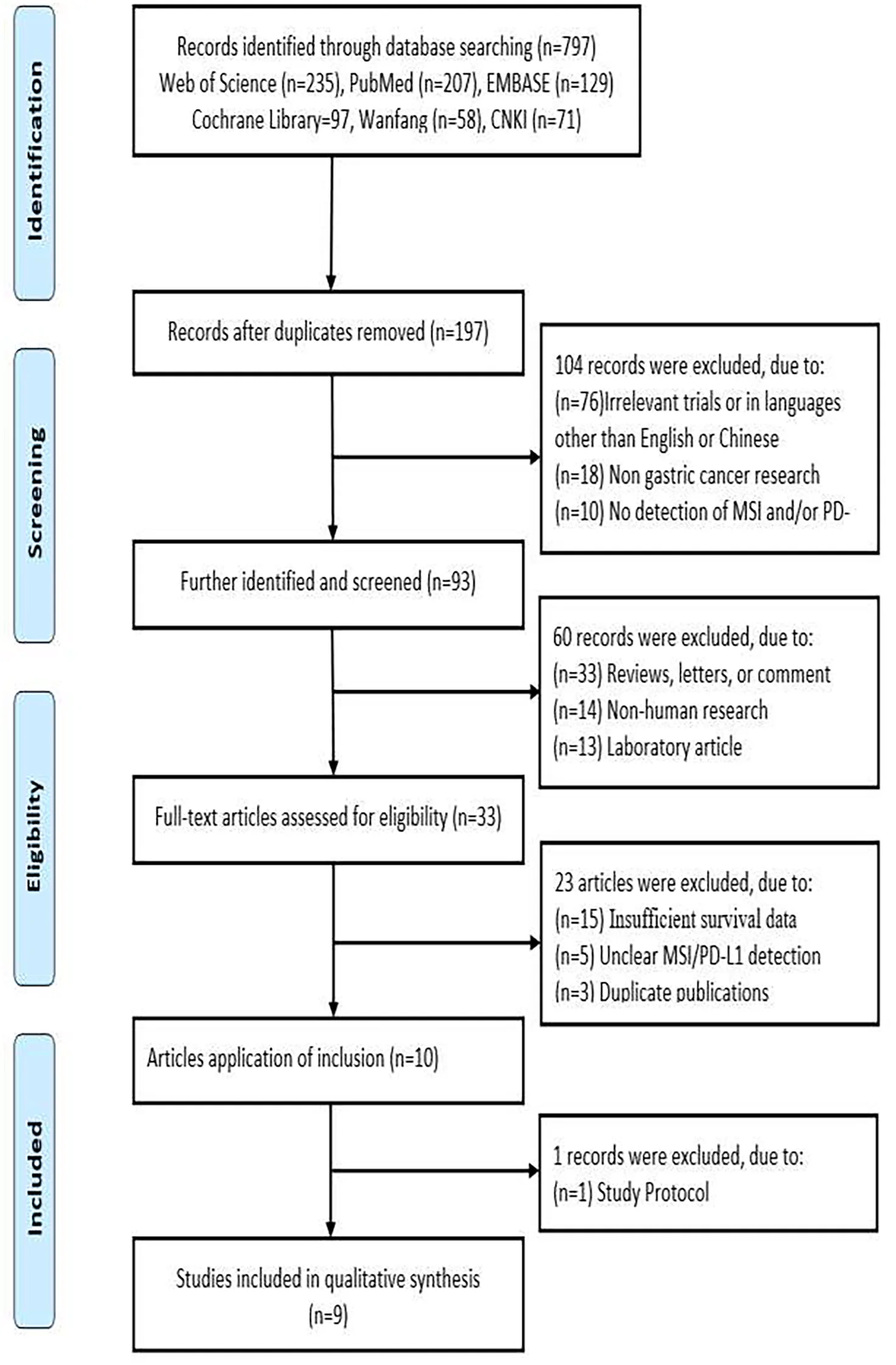
Study screening and selection flowchart.

**Table 1:** The main characteristics included in the study.

Study ID	Country /region	Study design	Sample size (n)	MSI detection method	MSI-H diagnostic criteria	PD-L1 detection antibody	TNM stage distribution (I-II/III-IV, n)	Lauren classification (intestinal/diffuse/mixed, n)	Main treatment regimen	Quality assessment^†^
Bartolomeo 2020 (4)	Italy	RCT(ITACA-S trial)	256	PCR	≥2 unstable loci	SP142	77/179	121/114/21	Perioperative neoadjuvant chemotherapy	High Quality
Wang 2020 (16)	China	Retrospective Cohort	2341	IHC (MMR protein)	Loss of ≥1 MMR protein	SP263	1468/899	904/668/769	Postoperative adjuvant chemotherapy	High Quality
An 2020 (19)	South Korea	Retrospective Cohort	790	PCR	≥2 unstable loci	NR	188/602	293/497(D+M)	Postoperative adjuvant chemotherapy	Moderate Quality
Junttila 2020 (9)	Finland	Retrospective Cohort	122	IHC (MMR protein)	Loss of ≥1 MMR protein	E1L3N	178/234	48/70/4	Perioperative neoadjuvant chemotherapy	Moderate Quality
Li (2025) (6)	China	Prospective Cohort	1007	PCR	≥2 unstable loci	22C3	499/508	335/309/363	Postoperative adjuvant chemotherapy	High Quality
Pietrantonio (2019) (10)	Multicenter	Retrospective Cohort	1556	IHC (MMR protein)	Loss of ≥1 MMR protein	NR	381/1094	678/825(D+M)	Perioperative neoadjuvant chemotherapy	High Quality
Wang (2022) (8)	China	Retrospective Cohort	118	PCR	≥2 unstable loci	22C3	12/16^*^	14/10/4	Perioperative neoadjuvant chemotherapy	Moderate Quality
Meng (2021) (17)	China	Retrospective Cohort	100	IHC (MMR protein)	Loss of ≥1 MMR protein	NR	54/46	44/56/-	Postoperative adjuvant chemotherapy	Moderate Quality
Li (2021) (5)	China	Retrospective Cohort	377	PCR	≥2 unstable loci	SP142	107/270	114/155/108	Perioperative neoadjuvant chemotherapy	Moderate Quality

* MSI data; **^†^**The results of the literature quality assessment were based on the Newcastle-Ottawa Scale; NR, Not reported.

RCT, Randomized Controlled Trial; MSI, Microsatellite Instability; MSI-H, Microsatellite Instability-High; MMR, Mismatch Repair; PD-L1, Programmed Death-Ligand 1; CPS, Combined Positive Score; IHC, Immunohistochemistry; PCR, Polymerase Chain Reaction; D+M, Diffuse/Mixed.

### 3.2 Quality evaluation results

The quality of included studies was assessed using the NOS for cohort studies and the Cochrane Risk of Bias Assessment Tool for the RCT *post-hoc* analysis. Among the 9 studies, 4 were classified as high-quality studies (7, 12, 14, 15) and 5 as moderate-quality studies (9, 13, 16–18) (**Supplementary Table 1**). The RCT *post-hoc* analysis (14) showed low risk of bias in random sequence generation and allocation concealment, while blinding-related bias was unclear (complete blinding was challenging due to the characteristics of surgical and chemotherapeutic interventions). No selective reporting or other significant biases were identified in all included studies.

### 3.3 Pooled analysis of prognostic value

#### 3.3.1 Overall survival

##### 3.3.1.1 MSI-H/MSI-L vs MSS

Six studies reported OS data for the comparison between MSI-H/MSI-L and MSS groups. Pooled analysis using a random-effects model showed that MSI-H/MSI-L status was significantly associated with better OS (HR = 0.641, 95%CI: 0.437-0.939, *P* = 0.025) (**Table 2**, **Figure 2**).

**Table 2:** Main results of pooled HRs in meta-analysis.

Comparisons	Heterogeneity		Summary HR (*95%CI*)	*P-value*	Studies
	*I*²(%)	*P*			
OS					
MSI-H/MSI-L vs MSS	70.6	0.005	0.641 (0.437-0.939)	0.025	6
MSI-H vs MSS	0.0	0.660	0.500 (0.357-0.700)	<0.001	3
PD-L1 Positive vs PD-L1 Negative	47.0	0.129	1.517 (1.077-2.137)	0.017	4
MSI-H+PD-L1 Positive vs MSS+PD-L1 Negative	15.6	0.306	2.076 (1.238-3.479)	0.006	3
MSI-H+PD-L1 Negative vs MSS+PD-L1 Negative	0.0	0.894	0.380 (0.255-0.566)	<0.001	3
DFS					
MSI-H/MSI-L vs MSS	0.0	0.792	0.530 (0.402-0.698)	<0.001	3
MSI-H vs MSS	0.0	0.615	0.506 (0.360-0.710)	<0.001	2
PD-L1 Positive vs PD-L1Negative	86.7	0.006	1.189 (0.472-2.998)	0.709	2
Immunotherapy response					
MSI-H vs MSS	0.0	0.341	3.580(2.410-5.320)	<0.001	2
MSI-H+PD-L1 Positive vs MSI-H+PD-L1 Negative	11.0	0.289	4.100(2.980-5.650)	<0.001	2
MSI-H+PD-L1 Positive vs MSS+PD-L1 Positive	0.0	0.317	3.270(2.310-4.630)	<0.001	2

OS, Overall Survival; DFS, Disease Free Survival.

**Figure 2:**
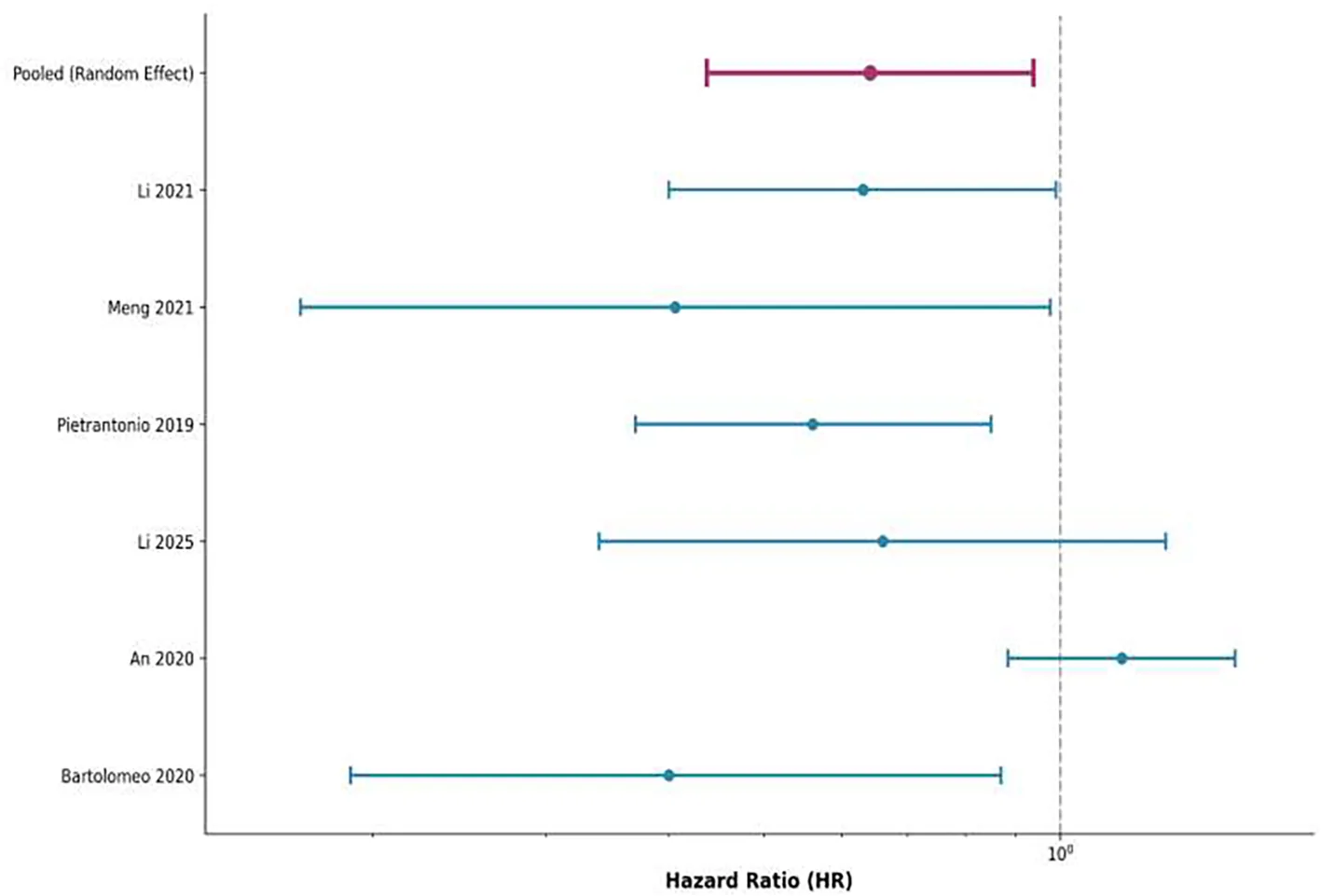
Forest plot of OS HRs: MSI-H/MSI-L vs. MSS subgroups.

##### 3.3.1.2 MSI-H vs MSS

Three studies provided OS data specifically for the MSI-H vs MSS comparison. Pooled analysis with a fixed-effects model revealed that MSI-H patients had substantially better OS than MSS patients (HR = 0.500, 95%CI: 0.357-0.700, *P* < 0.001) (**Table 2**). The absence of heterogeneity indicated high consistency in the prognostic value of MSI-H across these studies.

##### 3.3.1.3 PD-L1 positive vs PD-L1 negative

Four studies reported OS data for PD-L1 expression status. Pooled analysis using a fixed-effects model demonstrated that PD-L1 positivity was an independent adverse prognostic factor for OS (HR = 1.517, 95%CI: 1.077-2.137, *P* = 0.017) (**Table 2**).

##### 3.3.1.4 MSI-H+PD-L1 positive vs MSS+PD-L1 negative

Three studies reported OS data for this combined subgroup comparison. Pooled analysis with a fixed-effects model showed that MSI-H+PD-L1 positive patients had significantly poorer OS than MSS+PD-L1 negative patients (HR = 2.076, 95%CI: 1.238-3.479, *P* = 0.006) (**Table 2**).

##### 3.3.1.5 MSI-H+PD-L1 negative vs MSS+PD-L1 negative

Three studies provided OS data for this combined subgroup. Pooled analysis with a fixed-effects model revealed that MSI-H+PD-L1 negative patients had the most favorable prognosis, with significantly better OS compared to MSS+PD-L1 negative patients (HR = 0.380, 95%CI: 0.255-0.566, *P* < 0.001) (**Table 2**).

#### 3.3.2 Disease-free survival

##### 3.3.2.1 MSI-H/MSI-L vs MSS

Three studies reported DFS data for the MSI-H/MSI-L vs MSS comparison. Pooled analysis with a fixed-effects model showed that MSI-H/MSI-L status was significantly associated with improved DFS (HR = 0.530, *95%CI*: 0.402-0.698, *P* < 0.001) (**Table 2**), consistent with the OS findings.

##### 3.3.2.2 MSI-H vs MSS

Two studies provided DFS data for the MSI-H vs MSS comparison. Pooled analysis with a fixed-effects model demonstrated that MSI-H patients had better DFS than MSS patients (HR = 0.506, 95%CI: 0.360-0.710, *P* < 0.001) (**Table 2**), which was consistent with the OS results for this subgroup.

##### 3.3.2.3 PD-L1 positive vs PD-L1 negative

Two studies reported DFS data for PD-L1 expression status. Pooled analysis using a random-effects model showed no statistically significant difference in DFS between PD-L1 positive and negative patients (HR = 1.189, 95%CI: 0.472-2.998, *P* = 0.709) (**Table 2**).

#### 3.3.3 Subgroup analyses based on clinical and laboratory covariates

To explore sources of inter-study heterogeneity and identify effect modifiers of MSI prognostic value, we conducted subgroup analyses for the primary OS endpoint (MSI-H/MSI-L vs MSS) stratified by ethnicity, TNM stage, Lauren classification, sample size, study quality, MSI detection method and chemotherapy modality (**Table 3**). Interaction tests reached statistical significance for ethnicity, TNM stage, Lauren classification, MSI detection method and chemotherapy timing (all interaction *P* < 0.05). By contrast, subgroup interaction based on sample size (*P* = 0.200) and study quality (*P* = 0.680) was non-significant, indicating the prognostic impact of MSI-H remained stable regardless of cohort scale and study methodological rigor.

**Table 3:** Subgroup analysis of overall survival.

Subgroup dimension	Heterogeneity		Summary HR (95%CI)	P-value	Interaction P-value	Studies
	I²(%)	P				
MSI-H vs MSS/MSI-L						
Region/ethnicity					0.030	
Asian population	42.0	0.062	0.420 (0.300-0.590)	<0.001		4
European population	0.0	0.901	0.610 (0.380-0.980)	0.042		2
TNM stage					0.010	
Early stage (Stage I-II)	38.0	0.074	0.350 (0.220-0.560)	<0.001		3
Advanced stage (Stage III-IV)	45.0	0.058	0.580 (0.420-0.800)	0.001		3
Lauren classification					0.020	
Intestinal type-dominant	32.0	0.085	0.390 (0.250-0.610)	<0.001		3
Diffuse/mixed type-dominant	41.0	0.060	0.590 (0.430-0.810)	0.001		3
Sample size					0.200	
Large sample (≥500)	41.0	0.060	0.390 (0.250-0.610)	<0.001		3
Small sample (<500)	40.0	0.063	0.590 (0.430-0.810)	0.001		3
Study quality					0.680	
High-quality	35.0	0.078	0.460 (0.330-0.640)	<0.001		3
Moderate-quality	46.0	0.056	0.510 (0.340-0.770)	0.001		3
MSI detection method					0.009	
PCR-based assay	21.0	0.182	0.400 (0.270-0.600)	<0.001		3
MMR-IHC staining	30.0	0.136	0.690 (0.490-0.970)	0.032		3
Treatment modality					0.020	
Adjuvant chemotherapy + radical surgery	30.0	0.082	0.400 (0.280-0.570)	<0.001		3
Perioperative chemotherapy + radical surgery	0.0	0.910	0.620 (0.390-0.990)	0.045		3
PD-L1 positive vs PD-L1 negative						
PD-L1 antibody					0.038	
22C3 clone	0.0	0.910	1.830 (1.360-2.460)	<0.001		2
SP142/E1L3N clone	0.0	0.895	1.550 (0.640-3.760)	0.321		2

For pathological staging, the favorable prognostic impact of MSI-H/MSI-L was markedly more prominent in stage I-II early GC (HR = 0.350, *P* < 0.001) than stage III-IV advanced tumors (HR = 0.580, *P* = 0.001, interaction *P* = 0.010). Grouped by Lauren histotype, intestinal-type dominant cohorts presented more robust survival advantage associated with MSI-H/MSI-L (HR = 0.390, *P* < 0.001), whereas diffuse/mixed-type groups displayed attenuated protective effects (HR = 0.590, *P* = 0.001, interaction *P* = 0.020).

Grouping by MSI testing platform revealed significant methodological heterogeneity (interaction *P* = 0.009). Studies adopting multiplex PCR to distinguish MSI-H and MSI-L demonstrated stronger survival benefit for MSI-altered patients (HR = 0.400, *P* < 0.001), while cohorts relying on MMR-IHC exhibited weakened prognostic discrimination (HR = 0.690, *P* = 0.032). Grouping by chemotherapy timing also produced a significant interaction (*P* = 0.020): patients with MSI-H/MSI-L receiving postoperative adjuvant chemotherapy achieved superior survival outcomes compared with those treated with perioperative neoadjuvant chemotherapy (HR = 0.400 vs 0.620; *P* < 0.001 vs *P* = 0.045). Within all subgroups, *I*² < 50% and heterogeneity *P* > 0.05, demonstrating that grouping by these clinical and technical covariates substantially eliminated cross-study statistical heterogeneity.

For PD-L1 OS comparisons, antibody clone was an important source of heterogeneity (interaction *P* = 0.038). The 22C3 antibody group yielded a significant adverse prognostic effect of PD-L1 positivity (HR = 1.830, *P* < 0.001), while SP142/E1L3N cohorts showed non-significant OS correlation (HR = 1.550, *P* = 0.321).

### 3.4 Combined analysis of immunotherapy response

In the pooled analysis of immunotherapy response, two studies provided ORR data for pembrolizumab or nivolumab. For the MSI-H versus MSS comparison, showed that MSI-H patients achieved a significantly higher ORR than MSS patients (OR = 3.580, 95%CI: 2.410-5.320, *P* < 0.001). When further stratifying by PD-L1 status within the MSI-H group, two studies indicated that MSI-H+PD-L1-positive patients had a markedly superior ORR compared to MSI-H+PD-L1-negative counterparts (OR = 4.100, 95%CI: 2.980-5.650, *P* < 0.001). Lastly, in the subgroup of PD-L1-positive patients, two studies demonstrated that MSI-H+PD-L1-positive individuals also responded better than MSS+PD-L1-positive individuals, with an OR of 3.270 (95%CI: 2.310-4.630, *P* < 0.001).

### 3.5 Radar plot of pooled HR for survival

This radar plot visually presents the distribution characteristics of pooled HRs for OS and DFS of subjects across different subgroups stratified by molecular biomarker profiles in pooled analysis. MSI-related profiles were consistent protective factors for both OS and DFS, with similar effect sizes and heterogeneity characteristics across the two survival outcomes. PD-L1 positivity was only identified as a risk factor for OS but had no significant impact on DFS. Notably, a significant interactive effect of the combined MSI-H and PD-L1 expression status was observed on OS, where the positive combination acted as a strong risk factor and the negative combination as a strong protective factor (**Figure 3**).

**Figure 3:**
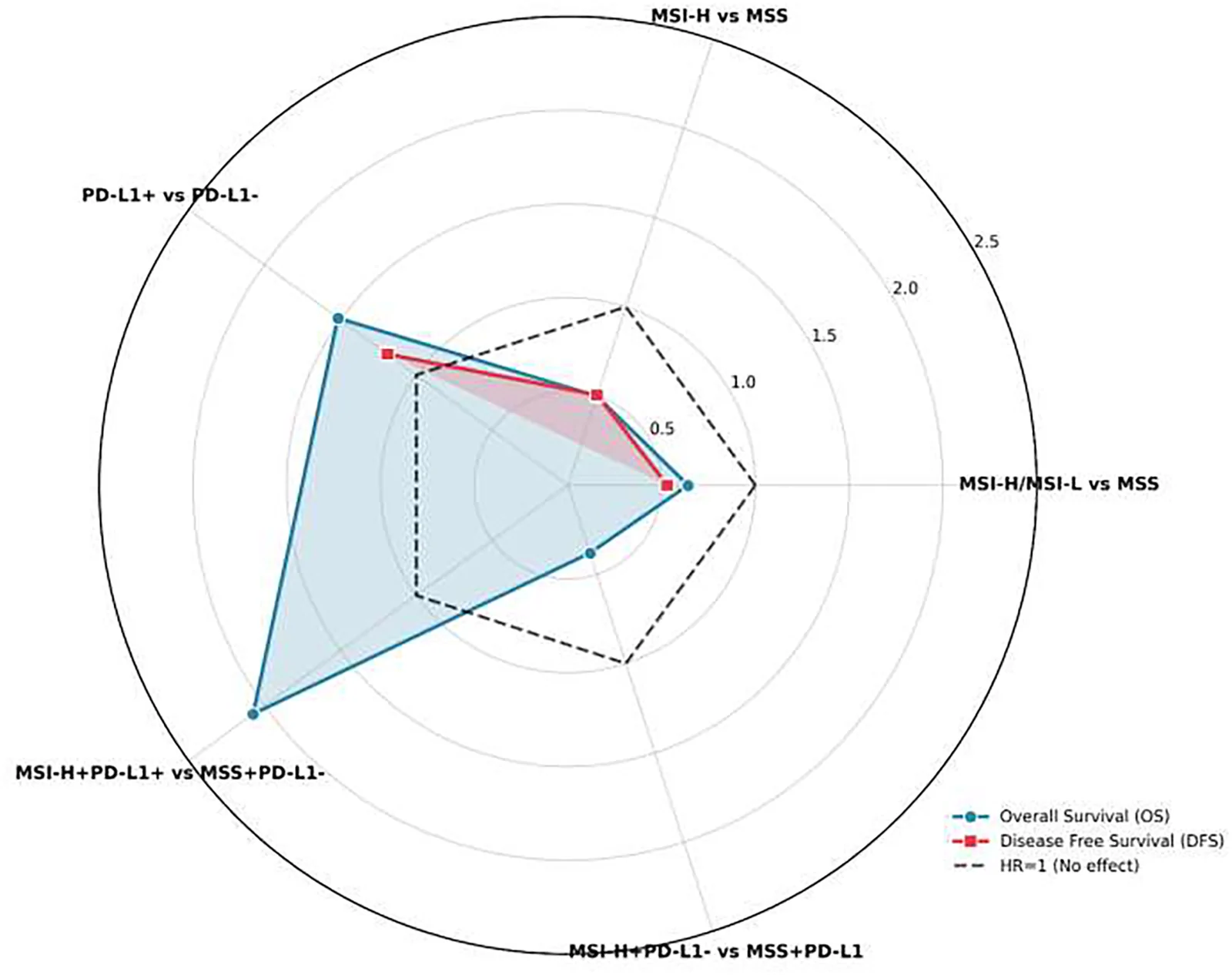
Polar plot of pooled HRs for OS and DFS across biomarker subgroups.

### 3.6 Sensitivity analysis and publication bias

Sensitivity analysis was performed by sequentially excluding each study, and the pooled HR/OR did not change significantly (**Supplementary Figure 1**), indicating that the results were stable and reliable. Publication bias detection showed that the funnel plots were symmetric (**Supplementary Figure 2**), and Egger’s test showed no significant publication bias (OS: *P* = 0.32; DFS: *P* = 0.28).

## 4 Discussion

This integrated analysis based on 9 studies involving 6,667 GC patients systematically evaluated the combined role of MSI and PD-L1 in GC prognosis and immunotherapy response. The results showed that MSI-H is a favorable prognostic factor for GC, while PD-L1 positivity is an adverse prognostic factor. The combination of the two markers can more accurately stratify patient prognosis, and MSI-H combined with PD-L1 positivity is a strong predictor of immunotherapy response.

### 4.1 Prognostic value of MSI status superiority of isolated MSI-H over MSI-H/MSI-L combination

Consistent with previous studies (20, 21), the present study confirmed that MSI-H GC patients have significantly better OS and DFS than MSS patients. For OS, the pooled HR of MSI-H alone vs MSS was 0.500 (*P* < 0.001), whereas the MSI-H/MSI-L combined group vs MSS showed a weaker protective effect (HR = 0.641, *P* = 0.025). This discrepancy highlights that MSI-H is the key driver of favorable prognosis, while MSI-L may act as a “confounder”, its inclusion dilutes the prognostic signal and increases inter-study variability. Mechanistically, MSI-H tumors harbor high mutation loads and abundant tumor-specific neoantigens, which activate robust anti-tumor immune responses by recruiting tumor-infiltrating lymphocytes (TILs) (22, 23). In contrast, MSI-L tumors have intermediate instability and limited immunogenicity, failing to elicit effective immune surveillance, thus explaining the superior prognostic utility of MSI-H alone.

For DFS, the consistency of MSI-related findings further validates its clinical reliability: both MSI-H alone (HR = 0.506, *P* < 0.001) and the combined MSI-H/MSI-L group (HR = 0.530, *P* < 0.001) showed significant DFS benefits, with no heterogeneity in either subgroup. This indicates that regardless of OS or DFS, MSI status (especially MSI-H) stably predicts a lower risk of recurrence and progression, which may be attributed to the less aggressive pathological features of MSI-H tumors (e.g., intestinal Lauren classification, fewer lymph node metastases) (19) and sustained immune-mediated tumor suppression.

### 4.2 PD-L1 expression serves as an adverse prognostic factor with heterogeneous survival endpoint effects

This study reveals a context-dependent prognostic role of PD-L1 expression in GC. For OS, PD-L1 positivity was an independent adverse prognostic factor (HR = 1.517, *P* = 0.017), consistent with most previous studies (24, 25). This is mechanistically driven by PD-L1’s ability to bind PD-1 on activated T cells, inducing T cell exhaustion and enabling tumor immune escape (8, 24). However, for DFS, no significant difference was observed between PD-L1-positive and negative patients (HR = 1.189, *P* = 0.709). This inconsistency may stem from two key factors: first, only 2 studies contributed to the DFS analysis of PD-L1, leading to insufficient statistical power to detect potential differences; second, variations in PD-L1 detection methods (antibodies 22C3, SP142, E1L3N) and cutoff value interpretations (e.g., CPS≥1 vs CPS≥10) across studies may have introduced measurement bias (26, 27). Additionally, DFS assessment relies heavily on postoperative follow-up intensity (e.g., frequency of imaging examinations), which may differ across research centers and further exacerbate heterogeneity.

### 4.3 Combined MSI and PD-L1 facilitates refined prognostic stratification for clinical practice

The most notable finding of this study lies in the prognostic stratification value of combined MSI and PD-L1 status. From the most favorable to the poorest prognosis, the OS ranking of combined subgroups was: MSI-H+PD-L1 negative (HR = 0.380, *P* < 0.001) > MSI-H alone (HR = 0.500, *P* < 0.001) > MSI-H/MSI-L combined (HR = 0.641, *P* = 0.025) > MSI-H+PD-L1 positive (HR = 2.076, *P* = 0.006). This hierarchical pattern underscores that PD-L1 expression acts as a critical “prognostic modifier” in MSI-H patients: while MSI-H inherently confers a survival advantage, PD-L1 positivity reverses this benefit, making MSI-H+PD-L1 positive patients have poorer outcomes than MSS+PD-L1 negative patients.

Mechanistically, this phenomenon likely reflects the dynamic state of the tumor immune microenvironment in MSI-H tumors. These tumors are typically characterized by a hot immune microenvironment rich in tumor-infiltrating lymphocytes. However, PD-L1 overexpression can suppress this antitumor activity by inhibiting T cell function (23). In contrast, MSI-H tumors that are PD-L1 negative retain intact T cell responses without immune checkpoint-mediated suppression, thereby achieving the most favorable prognosis. From a clinical perspective, this stratification addresses the limitations of single-marker testing. For instance, not all MSI-H patients have good outcomes; PD-L1 positivity can help identify those at higher risk who may require intensified adjuvant therapy. Conversely, in PD-L1 positive patients, MSI status helps distinguish those with relatively better prognosis (MSI-H and PD-L1 positive) from those with the poorest outcomes (MSS and PD-L1 positive).

### 4.4 Predictive value of concurrent MSI-H and PD-L1 positivity for immunotherapy response

Immunotherapy targeting the PD-1/PD-L1 axis has transformed the treatment landscape of advanced GC, and our findings further support the predictive value of combined MSI and PD-L1 status. As complemented by the immunotherapy response analysis, MSI-H patients had a significantly higher ORR to immunotherapy than MSS patients (OR = 3.580, *P* < 0.001), which aligns with the KEYNOTE-059 trial’s conclusion that MSI-H is a predictive biomarker for immune checkpoint inhibitor (ICI) efficacy (28). More notably, patients with both MSI-H and PD-L1 positivity achieved the highest response rates (OR = 4.100 vs. MSI-H and PD-L1 negative; OR = 3.270 vs. MSS and PD-L1 positive). This suggests that in MSI-H tumors, PD-L1 expression is not a negative predictor but rather a marker of immune sensitivity.

This apparent contradiction in prognostic outcomes arises as PD-L1 positivity correlates with poor OS alongside a high ORR. This discrepancy can be attributed to the distinct biological roles of PD-L1 across diverse contexts. From a prognostic perspective, PD-L1 expression signifies persistent immune escape and enhanced tumor aggressiveness. As a predictive biomarker, meanwhile, PD-L1 indicates a tumor immune microenvironment primed to respond to immunotherapy. Blockade of the PD-1/PD-L1 signaling pathway can effectively reactivate exhausted T cells and strengthen the systemic antitumor immune response (29). Clinically, this suggests that MSI-H+PD-L1 positive patients, despite their poor natural prognosis, are the most likely to benefit from immunotherapy. In contrast, MSI-H+PD-L1 negative patients may achieve durable survival with conventional therapy (e.g., surgery + chemotherapy) and may not require immediate immunotherapy intervention.

### 4.5 Sources of inter-study heterogeneity and immune/clinicopathological modifiers of MSI and PD-L1 prognostic effects

At the clinicopathological level, TNM stage and Lauren classification were significant modifiers (interaction *P* = 0.010 and 0.020, respectively). The protective effect of MSI-H/MSI-L was markedly stronger in stage I-II (HR = 0.350) and intestinal type (HR = 0.390), but attenuated in stage III-IV (HR = 0.580) and diffuse/mixed subtypes. Mechanistically, early intestinal type have lower burden and limited metastatic potential, allowing dMMR-driven intrinsic immunity to exert full antitumor effects; conversely, advanced diffuse type lesions foster an immunosuppressive microenvironment that partially offsets the survival benefit of MSI (7). Beyond pathological stratification, PD-L1 expression emerged as another critical modifier, yielding divergent MSI-related prognostic outcomes that require treatment-specific interpretation.

Our pooled analysis identified PD-L1 positivity as an independent adverse prognostic factor in resectable GC patients receiving radical resection plus perioperative chemotherapy alone. This finding, together with the established role of PD-L1 as a positive predictive marker for immunotherapy, explains the apparent discordance between our results and published immunotherapy trials (6, 30). Since all included cohorts excluded ICI-treated patients, the adverse effect under chemotherapy-only regimens is biologically plausible: membrane PD-L1 binds PD-1 on cytotoxic T lymphocytes, sustaining immune suppression, weakening antitumor surveillance, and accelerating progression, thus accounting for the poorer OS in PD-L1 positive subgroups (30). Conversely, when ICIs are administered, PD-L1 overexpression provides abundant targets for anti-PD-1/PD-L1 agents, reversing immune escape and improving response rates (6). Crucially, the adverse prognostic implication of PD-L1 from our pooled results should not be extrapolated to chemoimmunotherapy settings-a distinction rarely emphasized in prior GC meta-analyses.

Within MSI-H subgroups, patients with concurrent PD-L1 positivity had significantly worse survival than PD-L1 negative counterparts under chemotherapy alone. Beyond the PD-L1/PD-1 axis, multiple superimposed immune escape pathways drive this unfavorable phenotype (30, 31). First, dMMR triggers neoantigen accumulation and chronic inflammation, upregulating additional checkpoints (CTLA–4, LAG-3, TIM-3); cumulative suppressive signals exhaust tumor-infiltrating lymphocytes and counteract the high immunogenicity conferred by MSI-H (30). Second, sustained PD-L1 overexpression remodels the intratumoral immune landscape, causing CD8^+^ T-cell exhaustion and regulatory T-cell infiltration, which disrupts spontaneous antitumor immunity in the absence of ICIs (31). Third, chemotherapy alone cannot reverse these parallel inhibitory cascades; consequently, PD-L1 mediated survival disadvantage predominates despite the immunogenic background of MSI-H. This disadvantage is treatment dependent, with ICIs, concurrent MSI-H and PD-L1 positivity correlates with the highest objective response rates, underscoring that immunotherapy fundamentally reshapes the biological significance of this combined phenotype (6). Beyond clinicopathological and molecular immune modifiers, inter-study variations in MSI and PD-L1 detection methods introduced inherent methodological heterogeneity that may distort pooled estimates. The two mainstream techniques are not biologically equivalent: PCR-based MSI testing offers higher sensitivity for identifying microsatellite mutations and distinguishes distinct tumor subsets, whereas IHC for MMR proteins clusters dMMR and MSI-L lesions into a single category, potentially causing tumor misclassification across studies (7). Inconsistent use of PCR versus IHC across cohorts further amplifies heterogeneity and undermines the stability of pooled prognostic estimates.

### 4.6 Crosstalk between MSI/PD-L1 and classic gastric cancer molecular subtypes

GC exhibits profound molecular heterogeneity, wherein core driver biomarkers such as HER2 and CLDN18.2 dynamically interact with MSI and PD-L1 status to co-regulate the tumor immune microenvironment, clinical prognosis, and individualized therapeutic decision-making (32).

First, the correlation between MSI/PD-L1 status and HER2 amplification warrants attention. HER2 overexpressing gastric tumors generally harbor a low tumor mutational burden and weak intrinsic immunogenicity, and HER2 positivity is inversely associated with MSI-H status across GC cohorts (7). Activated HER2 downstream oncogenic signaling cascades transcriptionally upregulate PD-L1 expression on tumor cells, thereby synergistically reinforcing immune evasion. Clinically, the HER2+/MSI-H phenotype represents a rare dual-sensitive molecular subtype, amenable to both anti-HER2 targeted therapy and ICI. For HER2-amplified MSS tumors, anti-HER2 agents constitute first-line systemic therapy, whereas PD-L1 expression serves only as an auxiliary predictive biomarker for subsequent second-line immunotherapy upon disease progression (7, 33).

Second, the interaction between MSI/PD-L1 status and CLDN18.2 expression merits particular consideration. CLDN18.2 overexpression occurs independently of MSI status; thus, CLDN18.2 positive tumors may be either MSI-H or MSS. Upregulated CLDN18.2 remodels inflammatory infiltration within the gastric epithelium and moderately elevates cellular PD-L1 expression levels. Anti-CLDN18.2 targeted antibody therapy is applicable irrespective of MSI stratification. For CLDN18.2+/MSI-H patients, the combination of CLDN18.2 targeted therapy and immunotherapy exerts synergistic anti-tumor activity; conversely, CLDN18.2+/MSS patients derive limited benefit from immunotherapy alone, prioritizing CLDN18.2 directed regimens in this subset (32).

Collectively, MSI and PD-L1 belong to the category of immune-related predictive and prognostic biomarkers, whereas HER2 and CLDN18.2 represent driver oncogenic molecular markers. Their mutual crosstalk remodels the tumor immune microenvironment and guides stratified precision treatment. Concurrent multiplex detection of these four molecular markers is essential to refine prognostic stratification and formulate personalized therapeutic strategies for patients with GC.

### 4.7 Limitations

Several limitations of this study should be acknowledged. First, the subgroup analyses for certain combinations, as well as the DFS assessment for PD-L1 expression, were based on only two or three studies, which limits the statistical robustness of these specific estimates. Second, although all patients received radical surgery plus perioperative chemotherapy without immune checkpoint inhibitors, the inconsistent reporting of adjuvant versus neoadjuvant chemotherapy regimens across primary studies may have introduced residual confounding. Third, methodological heterogeneity arose from non-equivalent detection platforms; PCR-based testing distinguishes MSI-H from MSI-L, whereas MMR-IHC simply dichotomizes tumors as dMMR or pMMR, and variations in PD-L1 antibody clones and CPS cutoffs further contributed to measurement bias. Fourth, although age and sex data were extracted, subgroup analyses for these variables were precluded by heterogeneous reporting standards in the original literature. Fifth, most included studies were retrospective in design, carrying inherent selection bias, and the absence of immunotherapy-specific endpoints such as progression-free survival restricted our ability to evaluate treatment durability. Finally, given that all pooled data originated from patients treated without immunotherapy, our prognostic conclusions should not be extrapolated to chemoimmunotherapy or targeted therapy settings without further prospective validation.

## 5 Conclusion

In conclusion, this study confirms that MSI-H is a strong favorable prognostic factor for GC, while PD-L1 positivity indicates adverse OS prognosis. The combination of MSI and PD-L1 enables refined prognostic stratification, and MSI-H+PD-L1 positivity is a robust predictor of immunotherapy sensitivity. These findings provide evidence-based support for individualized treatment decision-making in GC patients and highlight the need for standardized molecular testing in clinical practice.

## Data Availability

The original contributions presented in the study are included in the article/**Supplementary Material**. Further inquiries can be directed to the corresponding author.
