# Supplementary material for: Microsatellite instability combined with PD-L1 expression in prognosis of gastric cancer: an integrated analysis based on individual patient data
Source: Front Oncol. 2026 Sep 17;16:1900024. doi: 10.3389/fonc.2026.1900024 (PMC13627007; doi:10.3389/fonc.2026.1900024)
Supplement: Supplementary file 1 [file Table1.docx]

Table S1 Included studies quality assessment.

| **Study ID** | **Study Type** | **Selection**  **(4 points)** | **Comparability**  **(2 points)** | **Outcome Measurement**  **(3 points)** | **Total Score** | **Quality Grade** |
| --- | --- | --- | --- | --- | --- | --- |
| Bartolomeo 2020[13] | Post-hoc analysis of clinical trial | ★★★★ | ★★ | ★★★ | 9 | High Quality |
| Wang 2020[14] | Retrospective cohort study | ★★★ | ★★ | ★★ | 7 | High Quality |
| An 2020[15] | Multi-cohort retrospective study | ★★★ | ★ | ★★ | 6 | Moderate Quality |
| Junttila 2020[10] | Clinical study | ★★★ | ★ | ★★ | 6 | Moderate Quality |
| Li 2025[11] | Retrospective cohort study | ★★★★ | ★★ | ★★★ | 9 | High Quality |
| Pietrantonio 2019[6] | IPD Combined analysis | ★★★★ | ★★ | ★★★ | 9 | High Quality |
| Wang 2022[16] | Retrospective cohort study | ★★★ | ★ | ★★ | 6 | Moderate Quality |
| Meng 2021[17] | Retrospective cohort study | ★★★ | ★ | ★★ | 6 | Moderate Quality |
| Li 2021[18] | Retrospective cohort study | ★★★ | ★ | ★★ | 6 | Moderate Quality |

Total score: 9 points (4 for Selection, 2 for Comparability, 3 for Outcome Measurement), 8 items in total.

Quality Grading Criteria (Aligned with AHRQ Standards).
